# Bird migration flight altitudes studied by a network of operational weather radars

**DOI:** 10.1098/rsif.2010.0116

**Published:** 2010-06-02

**Authors:** Adriaan M. Dokter, Felix Liechti, Herbert Stark, Laurent Delobbe, Pierre Tabary, Iwan Holleman

**Affiliations:** 1 Royal Netherlands Meteorological Institute, De Bilt, The Netherlands; 2 Swiss Ornithological Institute, Sempach, Switzerland; 3 Royal Meteorological Institute of Belgium, Brussels, Belgium; 4 Météo France, Toulouse, France

**Keywords:** bird migration, radar ornithology, weather radar, altitude profile, verification

## Abstract

A fully automated method for the detection and quantification of bird migration was developed for operational C-band weather radar, measuring bird density, speed and direction as a function of altitude. These weather radar bird observations have been validated with data from a high-accuracy dedicated bird radar, which was stationed in the measurement volume of weather radar sites in The Netherlands, Belgium and France for a full migration season during autumn 2007 and spring 2008. We show that weather radar can extract near real-time bird density altitude profiles that closely correspond to the density profiles measured by dedicated bird radar. Doppler weather radar can thus be used as a reliable sensor for quantifying bird densities aloft in an operational setting, which—when extended to multiple radars—enables the mapping and continuous monitoring of bird migration flyways. By applying the automated method to a network of weather radars, we observed how mesoscale variability in weather conditions structured the timing and altitude profile of bird migration within single nights. Bird density altitude profiles were observed that consisted of multiple layers, which could be explained from the distinct wind conditions at different take-off sites. Consistently lower bird densities are recorded in The Netherlands compared with sites in France and eastern Belgium, which reveals some of the spatial extent of the dominant Scandinavian flyway over continental Europe.

## 1. Introduction

Spatio-temporal information on bird migration is of invaluable use to scientists and society alike, but so far no sensor networks have been established that provide continuous and automated quantification of bird movements over large areas. Comprehensive monitoring of bird migration at continental scales can provide fundamental insight into migration patterns, the impact on migratory flight of synoptic scale factors like weather and orography and the selection of stop-over areas by migratory birds [1–3]. Strong potential exists for the applied use of continuous bird migration observations, for example in aviation for purposes of improving flight safety. In particular, military low-level flying has a high risk of en route bird strikes and spatial bird migration information is essential for generating reliable flight warnings to pilots. Other warning systems could be envisioned to reduce the collision risk of birds with wind farms, off-shore platforms and other man-made structures, by allowing people to predict and adapt to specific mass migration events.

Radar has had an immense impact on ornithology and the study of bird migration, because of its unique ability to monitor bird movements up to high altitudes and distances during both night and day [1,4–7]. Weather conditions [7,8], topographical features like coastlines [9,10] and orography [11] all influence migratory flight. The exact interplay of these synoptic scale factors and the migrational movements of birds is hard to study in general, mainly because of a lack of observational data.

**Figure 1.**
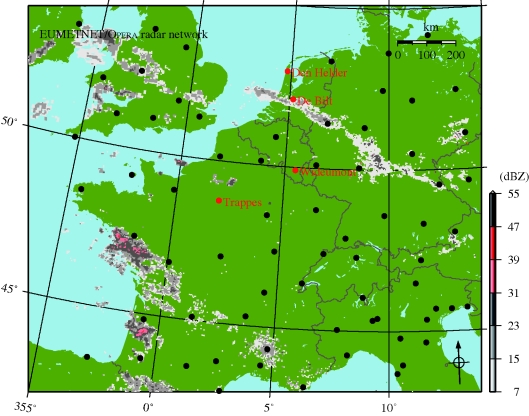
Map of operational weather radars for part of western Europe. Radar sites are indicated by bullets, the weather radars used in this study are labelled and coloured red. The Opera reflectivity composite is overlaid for 19 April 2008 19.30 UTC.

Operational weather radar networks exist in, for example, Europe and the United States for meteorological applications. These networks have a large areal coverage as illustrated in figure 1, showing part of the European network Opera (Operational Programme for the Exchange of weather RAdar information; [12]). Although designed for precipitation monitoring, weather radars can also observe biological scatterers. Boundary layer clear-air weather radar echoes are caused predominantly by arthropods (mostly insects) and flying birds [13–15]. If weather radar could detect and quantify aerial bird densities automatically, the existing weather radar network infrastructure could be used as a bird migration sensor network with an unprecedented coverage.

Although weather radars have been used in ornithological research for several decades [16], to our knowledge only two cases of continental bird migration were studied by a weather radar network [17,18], mainly because of the labour-intensive analytical work involved. To enable comprehensive monitoring of bird migration by weather radar networks, it is essential to (i) develop an automated and portable method for both the detection and the quantification of aerial bird densities and (ii) validate the reliability of this method with independent reference data. In this research article, we address both these requirements and report on the development and validation of a bird migration quantification algorithm, extracting altitude profiles of bird density, speed and direction. By applying the algorithm to a network of weather radars, we observed the progression of migratory flights along the migratory direction over a mesoscale range of several hundred kilometres.

## 2. Bird radar field campaigns

To obtain reference data for validating weather radar bird observations, we organized extensive field campaigns with a high precision dedicated bird radar. An ex-military pencil-beam X-band (*λ* = 3 cm) radar of the type ‘Superfledermaus’ [19] was stationed in the measurement volume of C-band weather radars in The Netherlands (19 August–16 September 2007), Belgium (18 September–22 October 2007) and France (10 March–9 May 2008; figure 1). The campaigns produced a large reference dataset consisting of bird densities and flight directions at 200 m altitude intervals and 1 h time intervals for a four-month period in total. The bird radar system has been well calibrated and makes use of state-of-the-art echo identification and quantification procedures [20,21]. For each detected echo wing beat patterns are automatically analysed, by which non-bird echoes like insects can be properly excluded. Bats cannot be automatically distinguished from birds, but are relatively less abundant in the study area [22], and, therefore, unlikely to have influenced measurements. The bird radar thus provides a high-quality reference for validating weather radar bird observations over a full migratory season. Bird radar measurement procedures are detailed in appendix A.

## 3. Bird detection and quantification by weather radar

Bird density quantification by weather radar relies on accurate methods of target identification. Bird-scattered signals need to be automatically distinguished from all other types of echoes, which include precipitation, ground echoes related to anomalous propagation, clear-air returns by insects and to some degree Bragg scattering from refractive index turbulence. Bragg scattering can be present especially at the top of the afternoon convective boundary layer [13,15], but is highly wavelength-dependent, appearing weaker on C-band by over 10 dBZ_e_ when compared with S-band [13,14].

Individual bird echoes can in principle be resolved by high-resolution weather radar [23], but usually the spatial resolution of operational base data is too coarse. Also, wing beat patterns cannot be recorded in operational scanning modes. Therefore, alternative methods of target identification need to be developed. At C-band bird-scattered signals are typically found at low reflectivity factors of −10 to 10 dBZ_e_ [24], but insects [13] and hydrometeors (i.e. water and ice particles) [25] can give rise to signals of similar strength. Additional information is necessary to separate out bird-scattered signals.

Central to our method of identifying bird-scattered echoes is an analysis of the radial velocity of the scatterers, which is measured routinely by Doppler weather radars. With the exception of strongly convective systems, the wind field carrying hydrometeors is spatially smooth and slowly varying, resulting in a low spatial variability of the measured radial velocities [26]. This also holds for Bragg-scattered echoes and echoes caused by insects, whose active flight tends to be slow and of which migration is predominantly windborne [27,28]. Bird migration gives rise to a much higher spatial variability in radial velocity [24,29–31], arising from individual variations in speed and direction of flying birds (see §4.1).

For most Doppler radars, an additional measure of radial velocity variance is available, namely spectrum width, which derives from the echo pulse statistics underlying a single resolution volume (instead of resolution-volume to resolution-volume variances). It has been suggested that spectrum width can be useful in target identification of various biological scatterers [32], but in this study spectral width was highly variable over different cases and meteorological conditions to make straightforward use of it in an automated algorithm. Nonetheless, there is potential for using spectrum width in bird detection algorithms, especially when using more sophisticated Doppler spectrum analysis that extends beyond the assumption of a Gaussian Doppler spectrum by most signal processors, which is often invalid for birds [24].

The developed bird detection algorithm produces a vertical profile of bird density, speed and direction every 5–15 min at 200 m altitude resolution, based on data at the 5–25 km range. At these close distances, the radar beam is sufficiently narrow to probe distinct altitudes, and range-dependent biases are largely avoided, which otherwise need to be compensated for [33]. Each extracted altitude profile is based on a spatial average over the 5–25 km range measurement window. For broad-fronted movement like most passerine migration [7], such an average will be representative, as migration will be spatially homogeneous. The algorithm is not designed to monitor very local migration features within the field of view of the radar. Detailed definitions are given in appendix A.

### 3.1. Bird detection

The algorithm is based on the existing wind-profiling algorithms for Doppler weather radars, using the volume velocity profiling (VVP) technique [26,34]. This technique was successfully applied in the context of bird migration profiling in a related study by van Gasteren *et al.* [30]. The VVP analysis delivers an altitude profile of the average speed and direction of the scatterers by fitting the data to a constant velocity model (see equation (A 1)). Additionally, at each height, a radial velocity standard deviation *σ*_r_ is calculated (see equation (A 2)). Recent studies have suggested that *σ*_r_ is a good discriminator between high-quality wind measurements and bird-contaminated wind profiles [24,30,31]. van Gasteren [30] showed that air layers with a high radial velocity standard deviation (*σ*_r_ > 2 m s^−1^) occurred at altitudes, where simultaneously bird migration was detected by an independent bird radar, which demonstrated that *σ*_r_ is also a good indicator for the presence of birds. Reliable bird density quantification could not be demonstrated in the study of van Gasteren [30] owing to contamination from residual precipitation and a large distance between the weather radar and bird radar sites (80 km). In §4.1, we validate *σ*_r_ as a discriminator for bird presence, where we will also discuss possible events unrelated to bird migration that can cause high values of *σ*_r_.

### 3.2. Removal of non-bird echoes

In addition to the *σ*_r_ criterion, we developed a target identification scheme to filter out non-bird echoes from the radar volume data. All reflectivity factors above 20 dBZ_e_ are masked as non-bird echoes, since such high values occurred rarely during bird migration in our study period and exceed reflectivity factors expected for broad-fronted passerine migration (20 dBZ_e_ corresponds to approx. 3500 passerine-sized birds per cubic kilometre at C-band (see equation (3.1) and figure 5 legend). For single-polarization Doppler weather radar, we developed a cell-finding algorithm that selects within each elevation scan contiguous areas above a certain reflectivity threshold (see appendix A).

**Figure 2.**
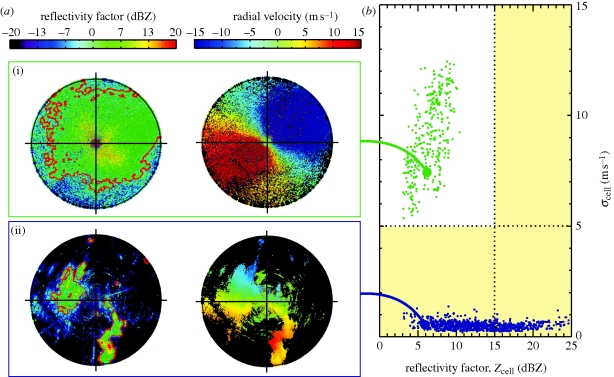
(*a*) Plan position indicators (PPIs) for reflectivity factor and radial velocity for a bird migration event ((i) 5 October 2007, 00.02 UTC) and an event with weak convective showers ((ii) 26 September 2007, 16.32 UTC). The PPIs show the 1.2° elevation scan up to 25 km range. Borders of identified reflectivity cells are indicated in red. (*b*) Radial velocity standard deviation *σ*_cell_ as a function of cell-averaged reflectivity factor *Z*_cell_ for reflectivity cells detected during intense bird migration events (green bullets, *σ*_cell_ = 9 ± 2 m s^−1^) and during events with convective showers (blue bullets, *σ*_cell_ = 0.5 ± 0.2 m s^−1^) for the Wideumont weather radar. Selected intense migration events were 4, 5, 6, 7 and 13 October 2007 from 17.00 to 09.00 UTC next day. Selected convective shower events were 24 September 2007, 9.00 to 18.00 UTC; 26 September 2007, 07.00 to 18.00 UTC; 17 October 2007, 06.00 to 17.00 UTC; 18 October 2007, 09.00 to 17.00 UTC. Only cells consisting of over 800 resolution volumes are shown to limit the number of scatter points. For the largest reflectivity cell in each PPI, a connecting solid line is drawn to its corresponding scatter point. Cells inside the yellow shaded segments (*σ*_cell_ < 5 m s^−1^ or *Z*_cell_ > 15 dBZ_e_) are classified as non-bird reflectivity cells and removed from the scan.

When selecting contiguous reflectivity areas, spurious precipitation cells may be detected in areas of intense bird migration as well. Examples of selected cells are given in figure 2, showing plan position indicators for a case of bird migration (figure 2*a* (i)) and precipitation (figure 2*b* (ii)). To identify selected cells in bird migration areas and discard them from the precipitation map, for each selected cell an average nearest neighbour variance *σ*_cell_ is computed for the radial velocities (see equation (A 3)). Analogous to *σ*_r_ discussed previously, the variance *σ*_cell_ is lower for wind-borne scatterers (both hydrometeors and insects) than for birds performing active flight. This difference is illustrated in figure 2*b*, where *σ*_cell_ is plotted as a function of the average cell reflectivity factor for a number of cells detected during intense bird migration events (green bullets) and cells detected during events with convective showers (blue bullets). A combined criterion using reflectivity and radial velocity information is used to assign cells to the precipitation map: cells with either a low variance or a high reflectivity factor (*σ*_cell_ < 5 m s^−1^ or *Z*_cell_ > 15 dBZ_e_) are removed from the data as non-bird scattering. From the remaining areas, an average reflectivity is calculated for each 200 m altitude interval.

We find that bird migration gives rise to a relatively high fraction of radial velocity dealiasing outliers (up to 15%) compared with precipitation (1%) and daytime clear air echoes [35] using the dual-PRF (pulse repetition frequency) technique. This is likely related to the fact that bird scattering arises from a few large complex targets for which the backscatter phase varies in time with slight geometry changes of the bird body. In the VVP analysis, radial velocity outliers are removed by an iterative fitting procedure. In the calculation of *σ*_cell_, the identification of outliers is less straightforward and has not been implemented. As a result, values of *σ*_cell_ for bird migration are typically higher than for *σ*_r_, and therefore also the optimal threshold in *σ*_cell_ is higher (5 m s^−1^) than for *σ*_r_ (2 m s^−1^).

One polarimetric weather radar was available in our study (i.e. in Trappes, France). By taking advantage of the additional information, especially the removal of precipitation from the data is improved. We exclude contiguous areas with a high correlation coefficient, *ρ*_HV_ > 0.9 (indicative of hydrometeors; [36]) or high differential reflectivity, *Z*_DR_ > 3.0 dB (indicative of insect; [37–40]). For polarimetric radar, the VVP analysis of radial velocity data remains necessary to determine the presence or absence of birds, because we found that the *Z*_DR_ criterion is often insufficient to filter out insect echoes, especially during cases with strong convective mixing.

### 3.3. Quantification of bird density

Bird density information can be obtained from reflectivity measurements, analogous to quantitative precipitation estimation. For S-band weather radars, empirical relationships have been found between reflectivity and the volumetric density of migrating birds [17,41], but only for a few preselected cases of bird migration in the absence of non-bird scatterers like precipitation. At a radar wavelength *λ*, the reflectivity *η*, equivalent reflectivity factor *Z*_*e*_ and bird density *ρ*_bird_ are related according to [25,41,42]3.1
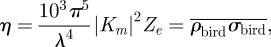
with *η* in cm^2^ km^−3^, *Z*_*e*_ in mm^6^ m^−3^, *K*_*m*_ = (*m*^2^ − 1)/(*m*^2^ + 2) with *m* the complex refractive index of the scatterers, *λ* (in cm) the radar wavelength, *ρ*_bird_ in birds per km^−3^ and *σ*_bird_ the bird radar cross section at C-band in square centimetres. The bar over the ‘*ρ*_bird_*σ*_bird_’ product denotes that this quantity is strictly an average over the different bird types or species *i* according to ∑_*i*_ *ρ*_bird,*i*_*σ*_bird,*i*_ and over the scanned viewing angles. With |*K*_*m*_|^2^ = 0.93 for water [25], we find using equation (3.1) that *η* is proportional to *Z*_*e*_ by a factor of 28.0 at S-band (*λ* = 10 cm) (see also [42]), 361 at C-band (*λ* = 5.3 cm) and 3.51 × 10^3^ at X-band (*λ* = 3.0 cm). Bird radar cross sections and therefore reflectivities *η* are of the same order of magnitude at these radar bands. However, because of the *λ*^−4^ proportionality in equation (3.1), birds cause much higher reflectivity factors *Z*_*e*_ in S-band compared with C-band (and much lower reflectivity factors in X-band).

Weather radar reflectivity can be converted to approximate bird densities by dividing *η* in equation (3.1) by an average bird radar cross section *σ*_bird_, which will be determined by the validation using bird radar measurements.

## 4. Results and discussion

### 4.1. Validation weather radar

**Figure 3.**
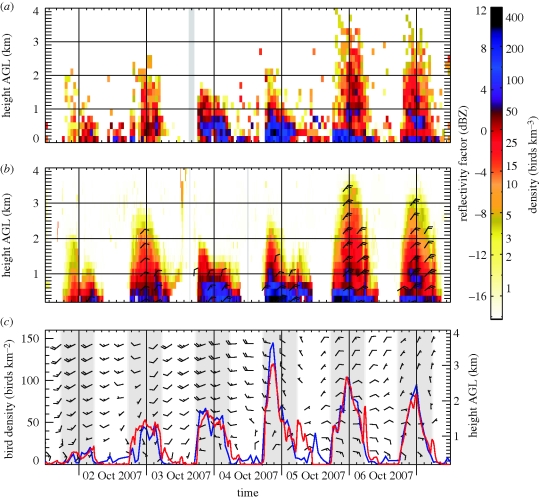
Comparison of the bird density altitude profiles determined by (*a*) bird radar and (*b*) weather radar. (*c*) Height-integrated bird densities are displayed for both weather radar (red) and bird radar (blue). Weather radar reflectivities were converted to bird density by assuming a constant weather radar cross section at C-band of *σ*_bird_ = 11 cm^2^ (see legends for figures 5 and 6). The period between sunset and sunrise is shaded in grey. Wind barbs in (*c*) show the wind profile from the Hirlam numerical weather prediction model. Each half barb represents 10 km h^−1^ and each full barb 20 km h^−1^.

In figure 3, we compare the bird density height profile determined by bird radar and weather radar for the period of 2–7 October 2007. A considerable nightly variance in flight altitudes and densities is recorded by the bird radar (figure 3*a*) during this period. While on 4 October, migration does not exceed 1.5 km, on 6 and 7 October, the bird radar records migration extending above 3 km height. These altitude profiles are closely reproduced by the weather radar algorithm (figure 3*b*). Also, a remarkable correspondence is observed between both sensors for the recorded absolute numbers of birds, as illustrated by the closely overlaying height-integrated bird densities (figure 3*c*).

For the quantitative verification, measurements were used from all three campaigns in De Bilt, Wideumont and Trappes, comprising 118 days of continuous data. We limit the quantitative verification of the weather radar algorithm to night-time hours only. During daytime bird flocks are common [7], which show no clear wing beat pattern and may therefore not be recognized by the automatic target identification of the bird radar. For this reason, the dawn ascent of daytime migrating birds is observed much more clearly by the weather radar than by the bird radar (e.g. on 5–7 October; figure 3). During night-time, the weather radar recorded above noise reflectivity factors in 61 per cent of the surveyed time–height layers (i.e. a specific height layer measured at a specific time) for airspace up to 4 km above ground level (AGL), while the bird radar recorded a non-zero bird density in only 38 per cent of the surveyed time–height layers. The dataset thus contains representative cases both with and without migrating birds.

#### 4.1.1. Bird detection

**Figure 4.**
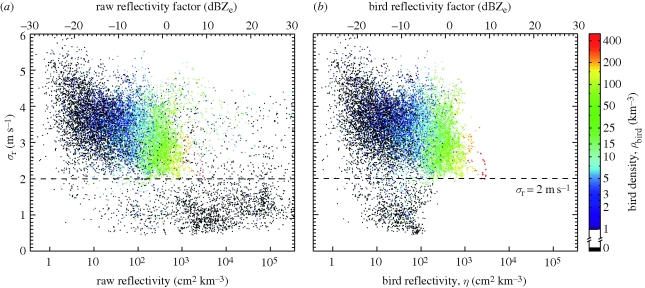
Radial velocity standard deviation *σ*_r_ as a function of raw reflectivity (*a*, reflectivity including non-bird echoes) and as a function of bird reflectivity (*b*, reflectivity cleaned from non-bird echoes). Each scatter point refers to a time–height layer, i.e. a specific height layer measured at a specific time. To limit the number of scatter points, only data for the Wideumont campaign are shown (22 September–21 October 2007, 14 742 detected non-empty time–height layers). In colour, the bird density is indicated as measured simultaneously by the bird radar. The large majority of non-zero bird densities are observed for time–height layers with *σ*_r_ > 2 m s^−1^.

Figure 4 shows for a large number of time–height layers the radial velocity standard deviation, *σ*_r_ (see equation (A 2)) as a function of raw reflectivity (figure 4*a*, non-bird echoes included) and as a function of bird reflectivity (figure 4*b*, non-bird echoes excluded). Bird densities as determined by the bird radar are indicated by colours. The large majority of non-zero bird densities are observed for time–height layers with *σ*_r_ > 2 m s^−1^, which confirms that a radial velocity standard deviation *σ*_r_ = 2 m s^−1^ successfully discards non-bird echoes. In figure 4*a*, a large number of points cluster around *σ*_r_ = 1 m s^−1^ at high raw reflectivity values, which can be attributed to precipitation events. A considerable number of time–height layers with *σ*_r_ > 2 m s^−1^ have obtained a high raw reflectivity from precipitation contaminations. The effect of removing these non-bird echoes on the reflectivity is shown in figure 4*b*. Precipitation-contaminated scatter points shift horizontally to lower reflectivity values, and high reflectivity gets always associated to high bird densities. Some residual precipitation contaminations remain in the region *σ*_r_ < 2 m s^−1^, while time–height layers for which all resolution volumes get assigned to the precipitation map are fully removed.

We define true bird presence when the bird density *ρ*_bird_ determined by bird radar exceeds 1 bird per km^−3^ and define a criterion for bird presence in weather radar data by *σ*_r_ > 2 m s^−1^. By collocating the bird radar and weather radar measurements, we can thus group all time–height layers into categories for the number of correct detections *H*, missed detections *M*, false detections *F* and correct non-bird detections *Z*. For the combined set of campaigns, we find that the weather radar system has a high probability of detection (POD) to be *H*/(*H* + *M*) (POD = 97%). The weather radar algorithm detects birds even at very low densities and bird migration events are unlikely to be missed. The false alarm ratio (FAR), *F*/(*H* + *F*), is however rather high (FAR = 42%), but the majority of these false alarms occur in a regime of very low bird reflectivity. Precipitation contaminations with reflectivity factors of −30 to −10 dBZ_e_ are frequent, but, as will be discussed below, such reflectivity factors correspond to very low bird densities below 1 bird per km^−3^ only. The rate of false detection steeply decreases with increasing bird reflectivity. Calculating the POD and FAR statistics for the subset of time–height layers with a reflectivity factor greater than −5 dBZ_e_, we find POD = 100% and FAR = 3% only. This regime of bird reflectivity factors is of most interest, as it corresponds to events with moderate to high bird densities greater than 10 birds per km^−3^.

Large radial velocity standard deviations, *σ*_r_, can also occur when the actual velocity field is not uniform (e.g. during strong wind shear; [43,44]) or when the terminal fall velocity is not constant (e.g. in a mixture of snow and rain; [45]). Such nonlinear wind fields may produce a high radial velocity standard deviation in the VVP analysis, which would falsely indicate an event of bird migration. Fortunately, nonlinear wind fields are usually associated with strong convection or frontal passage, which is often accompanied by precipitation. Precipitating areas will be removed by the precipitation masking algorithm, based on a high reflectivity factor (greater than 15 dBZ_e_) or a low value of *σ*_cell_. Since *σ*_cell_ is a locally calculated quantity over the nearest neighbours of each resolution volume, its value increases only owing to velocity variations over scales of up to a few kilometres and not by large-scale non-uniformities. Our bird radar measurements verify that most daytime weather radar echoes in clear air were caused by other scatterers than birds, which were correctly removed by the weather radar algorithm based on a low value of *σ*_r_. This confirms the suggestion by Caya & Zawadzki [43] that in clear air strong departures of the wind field from linearity are rare, at least during our verification period.

The high spatial variability in radial velocity of birds compared with wind-borne scatterers like insects and hydrometeors can be explained from the relatively sparse distribution of birds in airspace. When using close range data only (less than 25 km) up to bird densities of a few hundred birds per km^−3^, a radar resolution volume contains only one up to a few individual birds (assuming an even spatial distribution). The radar resolution is therefore sufficient to resolve part of the speed and directional variation between individual birds. In contrast, insect aerial densities tend to be much higher than bird aerial densities [28]. Each radar sample volume is thus more homogeneously filled and individual speed and directional variations of insects are averaged out. To some degree this smoothing effect is also observed for birds. As seen in figure 4, the radial velocity standard deviation *σ*_r_ slightly decreases when the bird density increases. A second cause of the high radial velocity variance observed for birds is their relatively fast active flight (10–25 m s^−1^; [46]), which allows for more variation in speed and direction within individuals than for insects, of which the airspeed is low [47].

#### 4.1.2. Quantification of bird density

**Figure 5.**
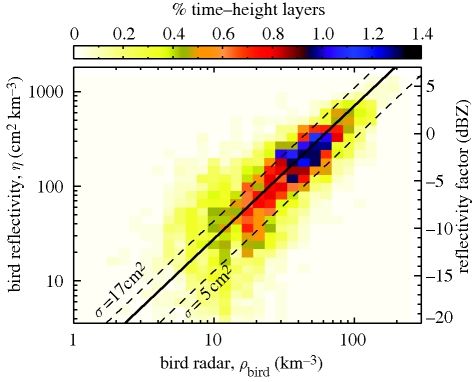
Correlation weather radar reflectivity and bird radar density in Wideumont, Belgium, corresponding to nightly (18.00–06.00 UTC) bird density estimates for 14 742 time–height layers recorded at 15 min time interval and 0.2 km height interval for the continuous period of 22 September–21 October 2007. We find *σ*_bird_ = 11 ± 6 cm^2^ and a correlation coefficient *R*^2^ = 0.73 (as calculated from a set of statistically independent bootstrap samples). Solid black line; best fit: *η* = 11*ρ*_bird_

**Figure 6.**
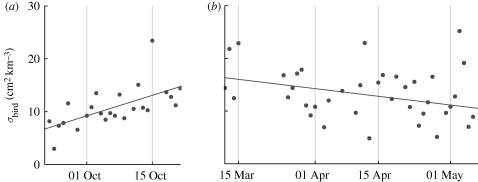
Seasonal trend in bird radar cross-section *σ*_bird_ at C-band. During autumn, the cross section increases in time (*a*, Wideumont campaign), while in spring, the cross section decreases (*b*, Trappes campaign). These trends reflect the increasing/decreasing proportion of larger bird species (mainly *Turdus* thrushes) during the migratory season.

In agreement with equation (3.1), we find a strong linear correlation between reflectivity *η* (measured by weather radar) and bird density *ρ*_bird_ (measured by bird radar), as illustrated in figure 5. An effective cross section for weather radar at C-band can be calculated as *σ*_bird_ = *η*/*ρ*_bird_ (see equation (3.1)). We find a median *σ*_bird_ of 11 ± 6 cm^2^, with seasonal trends shown in figure 6. Each data-point refers to a nightly average of *η*/*ρ*_bird_, omitting time–height layers with a low bird reflectivity (less than −5 dBZ_e_). From mid-September to mid-October (figure 6*a*), the average daily cross section is significantly increasing (*F*_22_ = 13.7, *p* < 0.002) from 7 to 15 cm^2^ by 0.3 ± 0.1 cm^2^ d^−1^. A reverse temporal trend is observed in spring when the cross section significantly decreases (*F*_41_ = 4.3, *p* < 0.05) from 16 to 10 cm^2^ by 0.1 ± 0.04 cm^2^ d^−1^ in the period mid-March to early May (figure 6*b*).

Wing beat data from the bird radar indicate that nocturnal bird migration during all field campaigns was strongly dominated by passerine migration. Seasonal changes in *σ*_bird_ must thus be explained from changes in the average body size of the migrating passerines. We can exclude insect scattering as a contribution to the observed cross-section changes, as the number of identified insect echoes by the bird radar decreased in autumn and increased over the course of spring, which are in fact the reverse trends that would explain the observed cross-section variations (background scattering by insects contributes to the measured reflectivity *η* by weather radar, but not to *ρ*_bird_ determined by bird radar, leading to an effectively larger *σ*_bird_). All large passerines (greater than 50 g; [48]) in western Europe are *Turdus* thrushes, with the exception of some species that are mostly daytime migrants like the European starling *Sturnus vulgaris* [5]. Both from visual observations, ringing data and radar observations [5,49,50], it is well established that the migration of *Turdus* species in western Europe increases steeply during October, while their return migration peaks relatively early in March. The increasing/decreasing abundance of large passerines in autumn/spring thus explains the seasonal changes in weather radar cross section.

For reasons of simplicity, we chose to use a constant bird radar cross section-*σ*_bird_ of 11 cm^2^ when converting weather radar reflectivity into bird density by equation (3.1).

During some nights in early September, larger effective *σ*_bird_ was observed up to 30 cm^2^ (data not shown). These cross sections are too large for the predominantly small passerines that migrate during this time of the season [49]. We may speculate that these events are related to migratory noctuid moths engaging in southward ‘return’ migrations [28,51]; however, dedicated measurements on the insect composition in the atmosphere would be necessary to confirm this. Because of the low migration intensities in early autumn, even a small insect background can have a relatively strong impact on the effective weather radar cross section. By mid-September, insect contamination no longer had clear impact on *σ*_bird_. During nocturnal spring migration, insect contamination is minor up to early June. Weather radar-extracted nocturnal bird densities drop below 5 birds per km^−3^ by mid-May at the end of the migration season. In spring, insect contaminations thus result in a bias of at most 5 birds per km^−3^; however, the contamination is likely less since by mid-May birds (e.g. Swifts) are still aloft and insect densities are much lower in March and April.

Although the aerial mass density of insects can be larger than for birds [28], scattering by birds is often enhanced relatively because of a larger radar cross section. For insects less than 1 cm, scattering enters the Rayleigh regime, where the radar cross section steeply decreases with the size of the object, while birds invariably give rise to a strong resonant (Mie) scattering. It will strongly depend on the typical insect size and densities whether insect contamination contributes significantly to the total measured reflectivity [52].

Differences in the representativity of the bird radar reference data and the weather radar data will contribute to the spread in the correlation between bird radar and weather radar in figure 5. An important difference between the two datasets is the size of the surveyed volume. For each time–height layer, the weather radar scans a 4 × 10^2^ km^3^ volume, while for the same time–height layer the fixed pencil beam of the bird radar surveys only a 0.01–0.1 km^3^ volume, depending on the altitude (see appendix A for the exact scanning strategies). Only in the case of ideal intense broad front migration [7] will the correlation between the two radars be unaffected by the discrepancy in surveyed volume.

### 4.2. Migration timing and altitude use

The effect of the environmental wind on flight altitudes becomes evident by comparing the bird density profile with the wind profiles calculated by the Hirlam numerical weather prediction model [53] (figure 3). In line with previous studies [54,55], we observe that birds adjust their flight altitude to make optimal use of tail winds along the predominant migratory direction (in this case towards southwest). For example, the Hirlam wind profile shows that on the night of 3–4 October wind conditions at low altitude were more favourable than at high altitude, where migrants would have encountered strong south-westerly head and side winds. On this occasion, migration did not extend above 1 km. On the other hand, on the night of 5–6 October, tail winds were present above 1 km and a large fraction of migration took place at high altitude.

Although decisions of birds to start migrating or not are primarily based on local weather conditions [8], we find that migration patterns observed at single sites can strongly depend on weather conditions elsewhere. This applies particularly to northern temperate climate, where frequent passage of high and low pressure systems causes a large spatial variability in weather. At individual sites, timing and altitude profile of bird migration was observed to be influenced by weather conditions at locations further up the migratory flyway. We will restrict our analysis to a spring migration event (19–20 April 2008), which illustrates the non-local character of bird migration. The general mechanism of flight altitude selection and the adaptive response of flying birds to changing meteorological conditions are beyond the scope of this manuscript.

We used four weather radars marked by red bullets on the map of figure 1. The Opera reflectivity composite is overlaid to give an impression of the synoptic weather situation. Active fronts related to a depression above the Bay of Biscay caused precipitation and easterly winds above southern France. A weak occlusion front at the Dutch–Belgian border slowly moved northward and diminished in activity in the course of the night.

**Figure 7.**
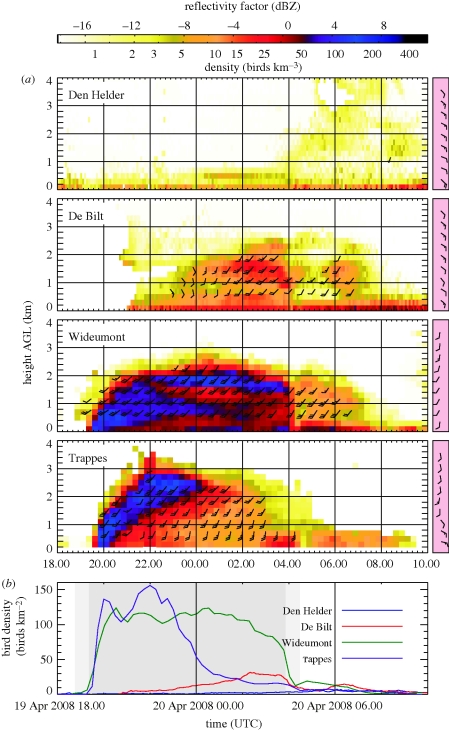
Bird densities as a function of time and altitude at different weather radar sites (figure 1) for the night of 19 April 2008; (*b*) shows the height-integrated bird densities over 0.2–4 km AGL. The period between sunset and sunrise is shaded in grey and civil twilight is shaded in light grey. The pink boxed inset on the right-hand side of each panel shows the Hirlam wind profile at 00.00 UTC. Bird flight speed and directions are indicated as barbs overlaid on the altitude profile. Each half barb represents 10 km h^−1^ and each full barb 20 km h^−1^. Around 00.00 UTC, a double-layered bird density profile is observed in Wideumont. We attribute the top band to birds that departed in the vicinity of Trappes, while the lower band results from birds departing more locally.

Figure 7*a* shows the retrieved bird density profiles for the four sites ordered from north (top) to south (bottom). Arrows indicating flight directions are overlaid with the bird densities, while barbs in the pink boxed insets show the Hirlam wind profile at 00.00 UTC. Large differences are visible between the sites, both in total density, timing and altitudinal profile of the migration.

Migration in Trappes is characterized by strong departure and ascent to high altitudes above 2 km, where birds experience favourable tail winds along the predominant migratory direction (according to our bird radar-tracking measurements during nocturnal migration towards 41° clockwise from north) as revealed by the wind profile. Low-altitude migration is avoided because of unfavourable easterly low-level winds. The migration intensity quickly drops after 00.00 UTC and the arrival of birds from southern latitudes is limited. The drop in bird density is related to the weather conditions south of Trappes, which, due to rain and easterly winds, were unsuitable for migration.

Migration in Wideumont (280 km north east of Trappes) starts out with strong departure at lower altitudes. Below 2 km, altitude tail winds are more favourable than above 2 km, where initially strong westerly winds prevail (wind data not shown). After 22.00 UTC, a remarkable double-layered bird density height profile is observed, with one band centred around 800 m AGL and a second band centred around 1900 m AGL. Since we started observing bird migration by weather radar in autumn 2007, such double-layered altitude profiles have been observed regularly during spring migration. The top altitude flight band appears after 4 h of flight time, which corresponds with a measured bird ground speed of 70 km h^−1^ to birds having travelled over a distance of about 280 km. These birds must therefore have departed in the vicinity of Trappes in northern France, where birds chose a flight altitude of 2500 m. Note that Wideumont is located 585 m mean sea level (MSL), therefore the 1900 m altitude band closely matches the cruising altitude after departure near Trappes. The double-layered density profile is not explained by the local wind profile in Wideumont. Rather, it is consistent with the migrants maintaining a constant flight altitude (above sea level) once they had completed the ascent phase of their migratory flight.

In De Bilt north of Wideumont, no birds depart in the early night. This is explained by the weak occlusion front slightly south of de Bilt, which blocks most migration. With this front weakening in activity, migration conditions become more favourable over the course of the night, and after 00.00 UTC, we do observe the passage and arrival of migrating birds. No migration is observed on the most northerly located weather radar in Den Helder.

Consistently lower bird densities are recorded in De Bilt and even more so in Den Helder compared with the sites in Trappes and Wideumont. We calculated migration traffic rates [20] defined as the number of migrating birds per hour crossing a 1 km broad vertical plane perpendicular to the migration direction at flight altitudes between 0.2 and 6 km. For nocturnal migration (between 18.00 and 05.00 UTC), seasonally averaged traffic rates are listed in table 1 during an autumn period (22 September–21 October 2007) and a spring period (15 March–15 May 2008). Ordering the radars according to their orthogonally projected distance from a line along the migration direction (towards 221° in autumn and 41° in spring as determined by the bird radar), we observe a northwest to southeast increase in the number of migrating birds. This increase reveals some of the spatial extent of the dominant Scandinavian migration flyway. We may expect many Scandinavian breeding birds to fly across Denmark and the southern tip of Sweden. Following their endogenous migration direction, birds will concentrate east of The Netherlands towards the centre of the flyway in Germany, eastern Belgium and France. Once data from the full Opera weather radar network become available, it will be possible to map for the first time the extent and dimensions of entire migratory flyways over Europe.

**Table 1.** Average nocturnal migration traffic rates (in birds km^−1^ h^−1^) during an autumn period (22 September–21 October 2007) and a spring period (15 March–15 May 2008) for the four radar sites shown in figure 1. Autumn data for Trappes were unavailable. Average bird ground speeds used in the calculation were obtained from the VVP radial velocity analysis. Both in autumn and spring, a northwest to southeast increase in the number of migrating birds is observed.

	migration traffic rate (birds km^−1^ h^−1^)			
season	Den Helder	De Bilt	Trappes	Wideumont
autumn 2007	243	504	—	857
spring 2008	85	170	515	562

## 5. Conclusions and outlook

We have developed an automated method for bird detection and bird density quantification by weather radar, extracting bird density, speed and direction as a function of altitude. The algorithm can run in near real-time with a time and altitude resolution of 5 min and 200 m, respectively, and was applied to four different Doppler weather radars in The Netherlands, Belgium and France. Generated altitude profiles are based on data up to 25 km range and thus provide a local characterization of the migration profile. The extracted weather radar bird densities have been validated with data from a high-accuracy dedicated bird radar, which was stationed in the measurement volume of different weather radars during the peak migration season of autumn 2007 and spring 2008. We find that Doppler weather radar is highly successful in detecting migrating birds and quantifying bird densities as a function of altitude. The probability of detection is (POD) is very high (99%), the false alarm ratio (FAR) is low (3%, provided that bird densities less than 10 birds km^−3^ are discarded) and weather radar reflectivity can be quantitatively correlated to the bird densities determined by the bird radar.

By applying our bird quantification method to a network of weather radars, we observed how mesoscale weather structured the intensity and timing of bird migration. In particular, the altitude profiles at individual sites were shown to be affected by the weather conditions at other locations. While the local wind field is an important variable for understanding local bird densities aloft, weather conditions in the vicinity of take-off sites can also strongly determine the observed flight altitude profiles.

The current study shows that automated bird migration monitoring by operational weather radar networks is feasible. The developed methods for bird detection and quantification can be easily extended to full operational weather radar networks. With the development of the Opera data centre for radar data within the coming 2 years [12], the establishment of a continent-wide bird migration sensor network in Europe is within reach. Besides better scientific understanding of continental bird migration, such a network can enable important applications both in aviation flight safety and environmental impact assessments for land use planning and development. Large-scale continuous monitoring by radar networks may improve our understanding of avian-borne disease spread, by revealing in more detail the temporal and spatial dynamics of migratory flyways, though we know of no examples of the use of radar in avian epidemiology so far [56].

Currently, polarimetric weather radar is rapidly becoming the new operational standard. Dual-polarization techniques will likely contribute to future improvements of the current bird migration quantification algorithm. We expect that automated bird migration quantification can also be implemented for weather radars operating at different radar wavelengths like S-band and X-band; however additional research and validation is required, because of the very different relative cross section of hydrometeors, insects and birds at these wavelengths. Weather radars are capable of providing spatial information on bird migration by using data from their full surveillance area [1,3,33]. A challenging task will be the development of automated methods to identify and quantify these spatial migration patterns at regional scales. Dedicated methods to identify (flocks of) large birds will be of interest to aviation, as these birds pose the highest collision risk.

For early-warning systems of bird migration, operational bird density forecasts are essential, which could be made in combination with spatially explicit bird migration models [57,58]. Such models may account for the inherently non-local character of bird migration and deal with the sparseness of radar observations, but will depend on data assimilation of areal bird density information. In meteorology, the integration of models and observations has become common practice and has greatly improved weather prediction. We expect that a similar synergy between bird migration observations by weather radar and migration models will greatly improve the description and predictability of bird movement.

## Appendix A. materials and methods

### A.1. Weather radar methods

The weather radars used in this study were located in Den Helder, The Netherlands (52.954 N/4.791 E, 51 m MSL), De Bilt, The Netherlands (52.103 N/5.179 E, 44 m MSL), in Wideumont, Belgium (49.915 N/5.505 E, 592 m MSL), and in Trappes, France (48.775 N/2.009 E, 191 m MSL). Typical C-band weather radar technical parameters are listed in [31]. Range/azimuth resolution equalled 1 km/1°, 1 km/1°, 250 m/1° and 240 m/0.5° for the four radars, respectively. All available Doppler scans were used in the analysis, i.e. depending on the radar from 8 to 13 scans distributed between 0.4 and 25° elevation.

For 200 m height intervals up to 6 km AGL, we approximate the local velocity field by a spatially constant model. The components of the local velocity field in the *x*-, *y*- and *z*- directions are approximated by constants *u*_0_, *v*_0_ and *w*_0_. The radial velocity *V*_r_ is then given by

A 1

with *θ*, *ϕ* the elevation and azimuthal angle, *u*_0_ and *v*_0_ the Cartesian ground speed components and *w*_0_ the vertical speed [34,45]. After a first least-squares model fit to equation (A 1), points with a deviation from the model of over 10 m s^−1^ are removed as outliers resulting from dealiasing errors [26]. Final velocity parameters are determined by a second model fit. From the fit residuals for each resolution volume *i* of the radial velocity data to equation (A 1), we determine the VVP retrieved radial velocity standard deviation *σ*_r_ in a least-squares sense:

A 2

where *V*_*r,i*_ are the observed radial velocities, *N* is the number of data points and *M* is the number of estimated parameters in the radial velocity model (*M* = 3 in equation (A 1)). Height-intervals with data gaps along the azimuth larger than 45° are rejected [26], which requires migration within the measurement window to be broad-fronted up to a certain degree. Data points *N* are all resolution volumes with an above-noise reflectivity (including those assigned to the precipitation map), excluding gates identified as clutter (see below).

For single-polarization Doppler radar data, contiguous cells of reflectivity are detected by a cell-searching algorithm [59], which defines cells as groupings of resolution volumes within an elevation scan for which each resolution volume has a reflectivity factor greater than 0 dBZ_e_ and at least five directly neighbouring resolution volumes that also meet this requirement. We find that a minimum threshold of 0 dBZ_e_ cuts out nearly all precipitation if an additional fringe of 3 km width is added around the selected cells to remove the low-reflectivity borders of precipitation areas. Cells within elevation scans are analysed by a cell-averaged nearest neighbour radial velocity standard deviation *σ*_cell_, according to

A 3

where the brackets 〈…〉_neigh_ denote an average over a resolution volume and its eight direct neighbours and 〈…〉_cell_ denotes an average over all resolution volumes assigned to a cell.

For dual-polarized Doppler radar, we make combined use of dual-polarization and Doppler quantities. Areas consisting of non-bird scatterers are removed based on a high cross-polar correlation coefficient *ρ*_HV_ > 0.9 or a high differential reflectivity, *Z*_DR_ > 3.0. Height layers with low radial velocity standard deviations *σ*_r_ < 2 m s^−1^ are rejected like in single polarization Doppler radar, which discards insect echoes for which dual-polarization moments often overlap with those of birds.

To exclude spurious echoes owing to ground clutter, static clutter maps were generated for each weather radar by performing a reflectivity average over several clear-air days outside the bird migration season for each resolution volume. All resolution volumes with a reflectivity average above −10 dBZ_e_ during these days are rejected permanently. A dynamic clutter map was implemented that excludes resolution volumes with a Doppler velocity in the interval [−1,1] m s^−1^, which filters out most echoes related to anomalous beam propagation [25].

### A.2. Bird radar methods

The bird radar has been stationed within the measurement volume weather radars in De Bilt, Wideumont and in Trappes. Hourly migration traffic rates were determined at the airfield of Soesterberg (52.13 N/5.28 E, 10 m MSL), the airfield of St Hubert (50.03 N/5.44 E, 577 m MSL) and at Flins sur Seine (48.58 N/1.52 E, 59 m MSL) at 6, 12 and 24 km distance from the respective weather radar sites.

Fixed beam measurements [20] were carried out at three elevation angles (5.6°, 22.5°, 79°), which allowed a good coverage of all flight altitudes up to 6 km. The beam was directed towards WNW (293°), thus perpendicular to the main migratory direction. During night-time, the three elevations were monitored every half hour between 17.00 and 05.00 UTC, and for the rest of the day every hour. Detection range was restricted to 7.5 km and recording time for a single elevation was 4 min. Echoes showing wing beat patterns corresponding to birds were automatically selected by a ‘vector support machine’ [21], which was trained based on a large sample of visually classified targets by an expert.

Between fixed beam measurements, the radar was operated in tracking mode and tracks of single targets were recorded for at least 20 s to determine flight paths. During the night, this was performed by an automatic search algorithm randomly selecting targets from all relevant heights. During daytime, tracking was performed manually with an operator selecting targets on the radar screen and an observer watching through a telescope mounted parallel to the radar antenna. Tracked targets were identified by the observer in the best case to species level.

Bird densities are calculated from the number of recorded echoes, the average bird ground speed vector and the calibrated bird- and aspect-specific surveyed volume [20].
